# A microbial community that alters mitochondrial morphology and age-related motor function in *C. elegans*

**DOI:** 10.1016/j.isci.2025.114128

**Published:** 2025-11-19

**Authors:** Nathan Dennis, Mireya Vazquez-Prada, Laura M. Freeman, Feng Xue, Lisa-Jane White, Antonis A. Karamalegos, William G. Sullivan, Brigita Kudzminkaite, Ian Brown, Jennifer R. Hiscock, Marina Ezcurra

**Affiliations:** 1 School of Natural Sciences, University of Kent, Canterbury CT2 7NJ, UK

**Keywords:** Molecular biology, Microbiology, Microbiome

## Abstract

Across diverse taxa, the composition of the microbiota is associated with host fitness. A mechanistic understanding of how microbial communities influence host physiology could lead to microbiota-based interventions for lifelong health. Here, we have developed a host-microbiota model system consisting of the model organism *C. elegans* combined with a defined natural microbiota (DefNatMta) consisting of 11 bacterial strains isolated from wild *C. elegans* to study natural host-microbiota interactions in the laboratory. We show that DefNatMta persists in the *C. elegans* gut, forming a stable and distinct gut microbiota. Utilizing this host-microbiota system, we find that DefNatMta affects age-related motility and protects against age-related decline in motor function. DefNatMta acts by altering metabolism and mitochondrial network dynamics in muscle and requires dynamin-related protein 1 (DRP-1), a regulator of mitochondrial fission to protect against age-related motility decline. Our findings are consistent with microbe-mitochondria communication affecting age-related muscle function.

## Highlights

• Host-microbiota model consisting of *C. elegans* and a defined natural microbiota (DefNatMta)
• DefNatMta forms a stable, distinct community in the *C. elegans* gut
• DefNatMta protects against age-related decline in muscle function
• DefNatMta alters mitochondrial networks and bioenergetics in muscle

## Graphical abstract

**Figure:**
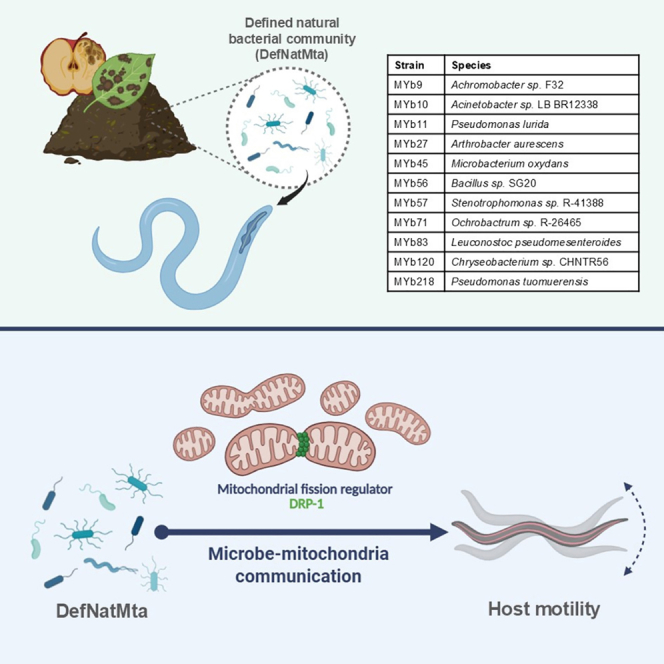

## Introduction

The microbiota, the community of microorganisms that live in a certain environment, forms a complex ecosystem and can impact metabolism and homeostasis of the host. Across diverse taxa, the microbiota is associated with host development, metabolism, and health.^1^^,^^2^^,^^3^ The microbiota in the gut has significant influence on the health and fitness of their hosts and is a key factor in multiple functions such as energy/nutrient absorption, immunity, intestinal permeability, hormone biosynthesis, and vitamin production. Gut microbes produce a vast number of enzymes and bioactive products that can influence health; some are beneficial, but others have toxic effects.^3^ Consequentially, alterations in the composition of the microbiota can lead to signaling to host tissues, contributing to altered host metabolism, dysregulated bodily functions, diseases, and impact on lifelong health.^4^^,^^5^^,^^6^^,^^7^

The mechanisms by which host-microbiota interactions affect the function of different tissues across life stages are not well understood. This is, in part, due to the high level of complexity of the human microbiota, consisting of hundreds of species altered by diet, lifestyle, and genetics.^8^ Species in the human microbiota are often anaerobic, difficult to cultivate, and require growth conditions only found in the gut, further limiting a mechanistic understanding.^9^ Laboratory studies using mice have improved the understanding of causative relationships between the microbiota and host metabolism and health, often involving use of germ-free mice and requiring costly and labor-intensive specialized facilities and equipment.^3^ The use of simple animal models, e.g., invertebrates with low-diversity microbiomes, offers cost- and time-efficient approaches for fundamental discoveries of host-microbiota interactions.^10^

*C. elegans* is a simple tractable model organism that offers informative and cost-effective routes for fundamental discoveries in biology. *C. elegans* is a bacterivore in which bacteria can serve both as food and microbiota.^11^ In standard laboratory conditions, *C. elegans* is maintained on a single bacterium, the domesticated *Escherichia coli* strain OP50, which *C. elegans* digests for nutrients, while other microbes are routinely removed through a bleaching protocol.^12^ As a result, the majority of *C. elegans* studies have ignored interactions between *C. elegans* and its microbiota. In its natural habitat, decomposing plant material, *C. elegans* are exposed to complex microbial communities, which can influence the physiology, development, immunity, and lifespan of *C. elegans*.^11^^,^^13^^,^^14^ These microbial communities have been described in several studies, paving the way to investigate *C. elegans* in the presence of its natural microbiome.^15^^,^^16^^,^^17^ The many advantages of *C. elegans* as an experimental system offer a valuable approach to establish a mechanistic understanding of natural host-microbiota interactions.

To perform mechanistic studies of host-microbiota interactions affecting health and fitness, we have established a host-microbiota model using *C. elegans* combined with a simple microbial community, here called defined natural microbiota (DefNatMta). DefNatMta is based on previous work examining the gut contents of wild *C. elegans* samples isolated from compost, rotting fruit, and invertebrate vectors.^15^ The bacterial community consists of 11 bacterial isolates and includes representatives of the most abundant genera of the *C. elegans*’ native microbiome, reflecting its bacterial taxonomic diversity.^15^^,^^16^^,^^17^ The strains are aerobic and grow in standard laboratory conditions.

Here, we establish DefNatMta combined with *C. elegans* as a highly tractable system to study natural host-microbiota interactions and determine the underlying mechanisms. We show that specific microbiota species are enriched within the gut, consistent with colonization. DefNatMta sustains *C. elegans* growth, development, and reproduction and is suitable for laboratory cultivation. We show that DefNatMta affects motility in the host, reducing motility in young adults but protecting against age-related decline. We further demonstrate that DefNatMta alters mitochondrial networks and bioenergetics in muscle. DefNatMta requires dynamin-related protein 1 (DRP-1) a regulator of mitochondrial fission to protect against age-related motility decline. Our findings are consistent with microbiota-mitochondria communication affecting age-related muscle function.

## Results

### DefNatMta populates the *C. elegans* gut and forms a stable and distinct microbiota

**Figure 1:**
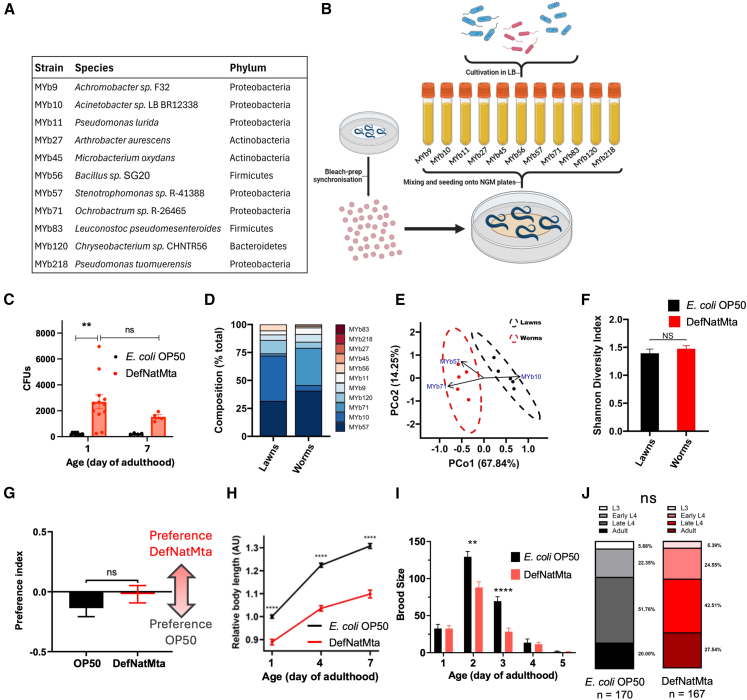
DefNatMta populates the *C. elegans* gut and sustains growth (A) Composition of DefNatMta with phylogenetic details. Bacteria were sourced from wild *C. elegans* samples isolated from compost, rotting fruit, or invertebrate vectors by the Schulenberg lab, see Dirksen et al. (2016). (B) Schematic of experimental setup. (C) CFUs recovered from the gut contents of animals fed with the DefNatMta or *E. coli* OP50. *n* = 150 per condition; pooled from nine biological replicates. Data are presented as mean ± SEM. (D) Strain-level composition of the DefNatMta lawn and the gut contents of DefNatMta-fed worms. *N* = 5 biological replicates. (E) Principal coordinates analysis of microbial diversity from DefNatMta lawns and DefNatMta-fed worms; PCoA was performed using Bray-Curtis dissimilarities. The ellipses represent the 95% confidence intervals, and the arrows represent the taxa significantly correlated with the ordination axes (*p* < 0.05; lengths scaled by R2). Correlations were assessed using the *envfit* function for *vegan* v.2.6–6.1. *N* = 5 biological replicates. (F) Shannon diversity indices of DefNatMta lawns and DefNatMta-fed animals. *N* = 5 biological replicates, data are presented as mean ± SEM and analyzed via Student’s *t* test. (G) Bacterial choice behavior of day 1 adult worms reared on either *E. coli* OP50 or DefNatMta. Indices >0 correspond to preference for the DefNatMta; indices <0 correspond to preference for *E. coli* OP50. *n* = 705 for DefNatMta, *n* = 1,164 for OP50; pooled from four biological replicates. Data are presented as mean ± SEM and analyzed via Student’s *t* test. (H) Body length of DefNatMta and *E. coli* OP50-fed animals, *n* = 90 per condition; pooled from three biological replicates. Data are presented as mean ± SEM. (I) Daily brood size of DefNatMta- and *E. coli* OP50-fed worms. *n* = 36 per condition; pooled from three biological replicates. Data presented as mean ± SEM. Reproductive span is the number of days with average daily individual brood counts >0 based on one-sample *t* test, which is 4 days for both conditions. Daily brood size analyzed via two-way ANOVA with post hoc false discovery rate (FDR)-corrected *t* tests. (J) Distribution of developmental stages (L3–adult) 60 h after egg synchronization. Developmental stages were classified as described in STAR Methods. *n* = 150 per condition; pooled from three biological replicates. Analyzed using Wilcoxon rank-sum test. Unless otherwise stated, data were analyzed using *t* tests. ∗∗∗∗*p* < 0.0001; ∗∗*p* < 0.01; ns, not significant (*p* > 0.05).

To characterize host-microbiota interactions in *C. elegans* we established a DefNatMta consisting of 11 bacterial isolates identified in wild *C. elegans* (Figure 1A). The bacterial isolates were selected based on previous work examining the gut contents of wild *C. elegans* samples isolated from compost, rotting fruit, and invertebrate vectors. This study identified 187 bacterial isolates belonging to 29 bacterial genera from the phyla Proteobacteria, Bacteroidetes, Actinobacteria, and Firmicutes and established an experimental microbiome consisting of 14 isolates.^15^ We constructed an overlapping, but not identical, microbial community consisting of 11 isolates, which we could grow in standard laboratory conditions to conduct comparative studies with the standard *C. elegans* bacterial source *E. coli* OP50. Eight of these were *Pseudomonas, Stenotrophomonas*, *Ochrobactrum*, *Acinetobacter*, *Leuconostoc*, *Chryseobacterium*, *Arthrobacter*, and *Achromobacter* isolates, falling within the 15 most abundant genera. Three additional genera belonging to abundant and taxonomically distinct bacterial orders of the *C. elegans*-native microbiome, *Achromobacter*, *Bacillus*, and *Microbacterium*,^15^ were also included. This defined microbiota contains some of the most abundant bacterial species found within wild *C. elegans* samples, constituting a taxonomically diverse community reflective of the native *C. elegans* microbiota.

To study the impact of this community on *C. elegans*, all bacterial isolates were cultivated individually in Luria Broth (LB) at 25°C, mixed in equal volumes, and seeded onto nematode growth media plates, using *E. coli* OP50 as control (Figure 1B). OP50 is widely used as the standard laboratory bacterial source for *C. elegans* cultivation but is a poor colonizer of young healthy *C. elegans* due to grinding of the bacteria by the pharynx and host immune responses that reduce bacterial load and proliferation in the intestine.^11^^,^^18^ We first verified whether DefNatMta populates the *C. elegans* gut by examining the number of viable cells recoverable from the gut contents of worms. On day 1 of adulthood, colony-forming units (CFU) from the gut contents of DefNatMta-fed worms were 2,687 CFU/animal, compared to 218 CFU/animal in OP50-fed worms, a 12-fold increase in gut bacterial load (Figure 1C). A significantly higher CFU count was also present on day 7 of adulthood (1,533 and 180 CFU/animal in DefNatMta- and OP50-fed animals, respectively), showing that in contrast to OP50, DefNatMta forms a stable bacterial community in the *C. elegans* gut. Next, we asked if the gut microbiota is compositionally distinct from the bacterial lawn on which *C. elegans* feeds. Such a difference would demonstrate enrichment of bacterial species and suggest bacterial colonization. We performed 16S rRNA sequencing of *C. elegans* gut contents and bacterial lawns. Our results demonstrated the formation of a selective gut microbiota, compositionally distinct from the bacterial lawn (Figure 1D). Principal coordinate analysis of Bray-Curtis dissimilarities confirmed this distinction (Figure 1E; Permutational multivariate analysis of variance [PERMANOVA], main effect of bacterial source: F_1, 9_ = 15.97, *p =* 0.006), indicating that the major taxa involved were *Ochrobactrum* MYb71 (highly enriched in the gut), *Acinetobacter* MYb10 (depleted in the gut relative to the lawn), and *Stenotrophomonas* MYb57 (moderately enriched in the gut), consistent with other studies showing intestinal colonization by MYb71 and MYb57.^15^^,^^19^ Despite the difference in microbial composition between the gut and the lawn, there was no change in Shannon diversity (an α-diversity index measuring richness and evenness) suggesting that composition, but not species diversity, is different in the gut compared to the external environment (Figure 1F). Consistently, Pielou’s evenness index also showed no significant difference between gut and lawn communities (Figure S1A), indicating that relative abundances are redistributed without affecting community evenness. From these findings, we conclude that DefNatMta forms a stable community within the gut, compositionally distinct from the bacterial lawn on which *C. elegans* feeds.

After establishing the formation of a unique gut microbiota, we examined the impact of DefNatMta on general health parameters to determine if DefNatMta is suitable for cultivation of *C. elegans* in the laboratory. DefNatMta slightly increased the feeding rate (Figure S1B; ANOVA, age × bacterial source interaction: F_2, 294_ = 5.818, *p* = 0.0033), and in a bacterial choice assay, animals showed preference for neither OP50 nor DefNatMta (Figure 1G), suggesting DefNatMta is not perceived as an inferior or pathogenic bacterial source. DefNatMta-fed worms exhibited a moderate reduction in body length that persisted throughout adulthood (Figure 1H; ANOVA, age × bacterial source interaction: F_2, 237_ = 10.31, *p* < 0.0001), along with a lower total brood size (Figure S1C), but no differences in reproductive span (4 days for both conditions; Figure 1I). Median lifespan was reduced by DefNatMta (12 days vs. 15 days; Figure S1D). DefNatMta had no impact on defecation rate (Figure S1E; ANOVA, age × bacterial source interaction: F_2, 116_ = 0.55, *p* = 0.5795; main effect of bacterial source: F_1, 58_ = 0.02, *p* = 0.8783) or intestinal barrier function (Figure S1F), indicating no major effects on intestinal physiology. DefNatMta had no impact on developmental timing (Figure 1J; Wilcoxon rank-sum test: W = 13323), indicating it provides adequate nutrition for growth to adulthood. Overall, these findings show that while DefNatMta has mild negative effects on reproduction and lifespan, it does not impair development or intestinal physiology and that DefNatMta is suitable for cultivation of *C. elegans* in the laboratory, providing an experimental system to study natural host-microbiota interactions.

### DefNatMta protects against age-related decline in muscle function

**Figure 2:**
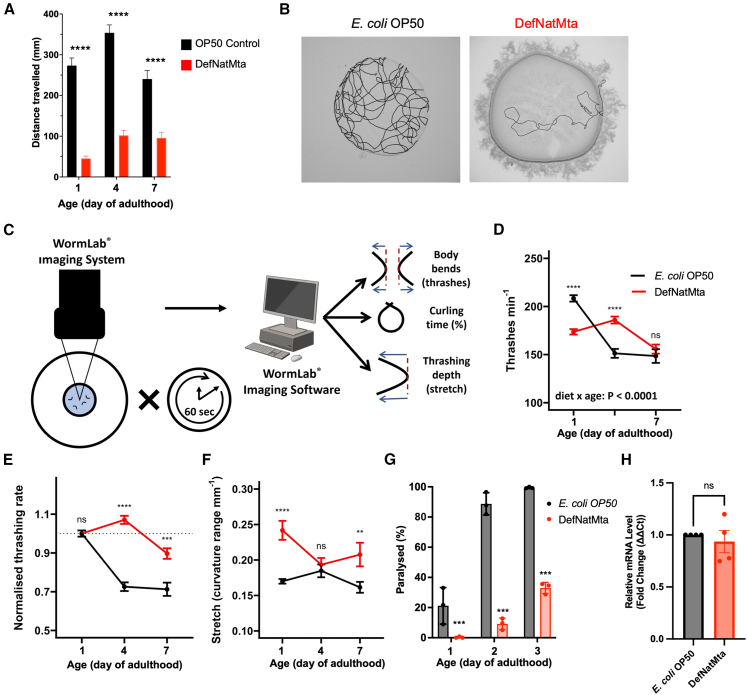
DefNatMta alters host motility and muscle function (A) Distance moved during a 4-h exploration assay. *n* = 135 per condition; pooled from three biological replicates. Data are presented as mean ± SEM and analyzed via two-way ANOVA with post hoc FDR-corrected Student’s t tests. (B) Representative images showing traced tracks of distance moved. (C) Schematic of motility assessment using the WormLab Imaging System and Software. (D and E) Raw (D) and day 1 adult-normalized (E) frequencies of lateral swimming (thrashing rate) of OP50- and DefNatMta-fed worms. *n* = 87 for DefNatMta, *n* = 86 for OP50; pooled from three biological replicates. Data are presented as mean ± SEM. (F) Thrashing depth (stretch). *n* = 90 per condition; pooled from three biological replicates. Data are presented as mean ± SEM. (G) Paralysis rate in GMC101 animals expressing Aβ1-42 in body wall muscle. *n* = 974 for DefNatMta, *n* = 947 for OP50; pooled from three biological replicates. Data are presented mean values with per-trial averages. (H) Aβ mRNA expression in GMC101 animals fed DefNatMta compared to that in animals fed *E. coli* OP50. Aβ mRNA expression was normalized to the expression of *cyc-1*. Error bars represent the standard error. Three technical replicates for each sample were used, and four biological replicates were analyzed. Bars represent the grand mean ± SEM. Each dot represents the mean value from a single trial. Statistical analysis performed using two-way ANOVA with post hoc FDR-corrected Student’s *t* tests (D–G) and Welch’s *t* test (H). ∗∗∗∗*p <* 0.0001; ∗∗∗*p <* 0.001; ∗∗*p <* 0.01; ns, not significant (*p >* 0.05).

After establishing the combined DefNatMta-*C. elegans* model system, we asked how DefNatMta affects foraging behaviors, as bacteria can affect foraging in *C. elegans*. While exploring a bacterial lawn, *C. elegans* adopts two major behavioral states: roaming, characterized by high locomotion speed and long-distance movement, and dwelling, defined by low speed and frequent reversals.^20^^,^^21^ Different bacterial strains elicit distinct roaming and dwelling behaviors in *C. elegans*, and *C*. *elegans* exhibit*s* enhanced roaming upon encountering aversive signals, e.g., from pathogens.^22^^,^^23^^,^^24^ We cultivated animals on OP50 and DefNatMta and measured the distance animals covered on the bacterial lawns during a 4-h period. Day 1 adults fed on DefNatMta roamed much less than OP50-fed animals, covering only 45 mm compared to 273 mm for the OP50 controls (Figures 2A and 2B). We examined if these differences persisted with age and measured distance traveled on days 4 and 7 of adulthood. At both time points, DefNatMta-fed animals continued to show reduced roaming behavior. On day 4, they traveled 102 mm, vs. 353 mm for OP50 controls, and on day 7, they traveled 96 mm compared to 240 mm (Figure 2A). We noted that DefNatMta altered the typical pattern of age-related changes in locomotion. OP50-fed animals showed a 29% increase between days 1 and 4, followed by a 32% decrease between days 4 and 7 (*p* = 0.0013 and *p* < 0.0001 respectively; two-way ANOVA), consistent with studies showing age-related changes in spontaneous movement.^25^^,^^26^^,^^27^ In contrast, DefNatMta-fed animals increased the distance moved by 125% between days 1 and 4 (*p* = 0.0317; two-way ANOVA) and then maintained a consistent distance between days 4 and 7 (Figure 2A).

A possibility is that physical (material) differences in bacterial lawn structure influences *C. elegans* locomotion, leading to reduced roaming in DefNatMta-fed animals. To test this, we measured the viscoelastic properties of OP50 and DefNatMta by oscillatory rheology. Measurements were conducted on bacterial lawns collected under the experimental conditions used for behavioral assays. Frequency sweep measurements identified a similar linear viscoelastic region for both bacterial sources, with storage (G′) consistently higher than loss (G″), confirming their viscoelastic nature (Figure S2A). Next, we directly compared the viscoelastic properties of OP50 and DefNatMta by measuring viscosity at shear rates of 0.1/s and 100/s. At 0.1 s^−1^, both OP50 and DefNatMta showed pronounced viscous resistance (2.1 × 10^7^ and 2.4 × 10^7^ mPa s, respectively), consistent with the rheological profile of a highly structured network or gel-like material. At 100 s^−1^, viscosity decreased similarly for OP50 (54,912 mPa s) and DefNatMta (44,495 mPa s), with no significant differences (Figure S2B). These data suggest that the motility effects are unlikely to arise from material properties of the bacterial lawns.

These findings are consistent with DefNatMta neither being a nutritionally inferior bacterial source nor physically hindering the movement of the animals. This led to us ask how host-microbiota interactions affect other measures of motility, as well as healthspan, which is the extent to which normal physiological function is retained with age. We focused on age-related motility, given that reduced gut microbiota diversity is linked to increased age-related frailty.^28^^,^^29^ Although DefNatMta negatively impacted lifespan, multiple studies suggest that loss in microbiome diversity is more closely associated with age-related frailty than with chronological aging itself.^30^^,^^31^ As with the exploration assay, we assessed movement during early to mid-adulthood, a stage marked by the most rapid decline in motor function, using a swimming assay, a crucial measure of locomotor behavior and muscle function combined with automated image analysis (Figure 2C). Similarly to previous studies showing age-related decline in locomotory rate and muscle tissue integrity,^26^^,^^32^ we found that in OP50-fed animals, young adults had a swimming rate of approximately 200 strokes per minute, which declined by 30% between days 1 and 7 of adulthood. DefNatMta-fed animals exhibited a 14.6% reduction in swimming rate on day 1 compared to OP50 controls, suggesting reduced motility in young animals. However, unlike OP50-fed worms, these animals showed a negligible decline in swimming rate between days 1 and 7 (Figure 2D; ANOVA, age × bacterial source interaction: F_2, 167_ = 25.69; *p <* 0.0001), retaining 90% of their day 1 adult thrashing rate at day 7 of adulthood (Figure 2E), suggesting DefNatMta protects against decline later in life. We also measured stretch, a measure of the average depth of swimming strokes^33^ (how forcefully the body bends). We found that stretch did not change with age in OP50-fed animals and was significantly increased by DefNatMta (Figure 2F; ANOVA, age × bacterial source interaction: F_2, 87_ = 0.87, *p* = 0.0208), particularly on days 1 and 7 of adulthood. Our findings are in agreement with previous work showing that thrashing rate in OP50-fed animals exhibits age-related decline, while stretch remains relatively stable.^33^ This suggests that although the frequency of movement decreases with age, the muscle contractile strength required to produce a full bend is retained longer. We interpret the increase in stretch in DefNatMta-fed animals as an indicator of altered muscle function.

Given that DefNatMta suppressed age-related motility decline while also reducing motility in young adults, we asked if DefNatMta promotes healthspan or simply induces a reduction in motility in early adulthood, which persists with age. We tested the effect of DefNatMta on proteotoxicity, the impairment of cellular function caused by the accumulation of misfolded or damaged proteins. Proteotoxic stress contributes to various age-related conditions, including sarcopenia, cardiomyopathies, and neurodegenerative diseases.^34^^,^^35^^,^^36^ We assessed if DefNatMta improves cellular function rather than just altering behavior utilizing a model of proteotoxicity expressing human amyloid-β (Aβ) in body wall muscle, resulting in Aβ aggregation and progressive age-related paralysis.^37^ Worms cultivated on OP50 exhibited more rapid age-related paralysis than those cultivated on DefNatMta (ANOVA, age × bacterial source interaction: F_2, 30_ = 58.64, *p* < 0.0001; Figure 2G), with total paralysis rates in OP50-fed worms reaching 96.5% and 100% on days 2 and 3 of adulthood, respectively. In contrast, paralysis rates in DefNatMta-fed animals reached just 9.6% and 34.7% on days 2 and 3 of adulthood, respectively. To determine whether differences in Aβ expression might underlie the observed protective effects, we quantified Aβ mRNA levels by RT-qPCR, normalizing to expression of *cyc-1,* encoding the ortholog of human cytochrome *c*1. Aβ transcript levels were nearly identical between DefNatMta-fed and OP50-fed worms (Figure 2H; 0.92-fold difference), indicating that protection is not explained by altered Aβ expression. Together, these findings suggest that DefNatMta leads to reduced motility in young animals, reduced age-dependent decline in motility, and increased age-dependent resistance to proteotoxic stress.

### DefNatMta alters host mitochondrial dynamics in body wall muscle

Locomotion in *C. elegans* is directly controlled by the body wall muscles, equivalent to mammalian skeletal muscle, which work with the nervous system to generate movement.^38^ As DefNatMta reduces swimming rate in young adults, we asked if DefNatMta alters muscle morphology or neuromuscular transmission. We quantified body wall sarcomere organization using fluorescently tagged myosin and actin^39^ but did not find any changes in muscle cell area or gaps between filaments^34^ (Figures S3A–S3I). Next, we asked if DefNatMta alters the decline in body wall sarcomere organization and neuromuscular junction function that occurs with age.^32^^,^^40^^,^^41^ Scoring of GFP-tagged myosin filaments revealed no difference in sarcomere organization associated with age or bacterial source (Figures S3J and S3K; ordinal regression, likelihood ratio test relative to a null model: χ^2^(5) = 9.90, *p* = 0.078). Similarly, assessment of filament density via electron microscopy did not indicate any differences between OP50- and DefNatMta-fed animals (Figures S3L and S3M; ANOVA, age × bacterial source interaction: F_1, 36_ = 0.45, *p* = 0.5045; main effect of bacterial source: F_1, 36_ = 2.84, *p* = 0.1005). Neuromuscular transmission efficiency was measured by quantifying contractability and response to touch stimuli,^42^^,^^43^ revealing no differences in contractibility (ANOVA, age × bacterial source interaction: F_3, 477_ = 0.68, *p* = 0.5640; main effect of bacterial source: F_1, 477_ = 0.07, *p* = 0.7897) or touch responsiveness (ANOVA, age × bacterial source interaction: F_3, 223_ = 0.01, *p* = 0.9984; main effect of bacterial source: F_1, 223_ = 1.64, *p* = 0.2016) between OP50- and DefNatMta-fed animals (Figures S3N–S3P). Together these findings indicate that DefNatMta alters muscle function independently of sarcomere organization and motor unit function.

**Figure 3:**
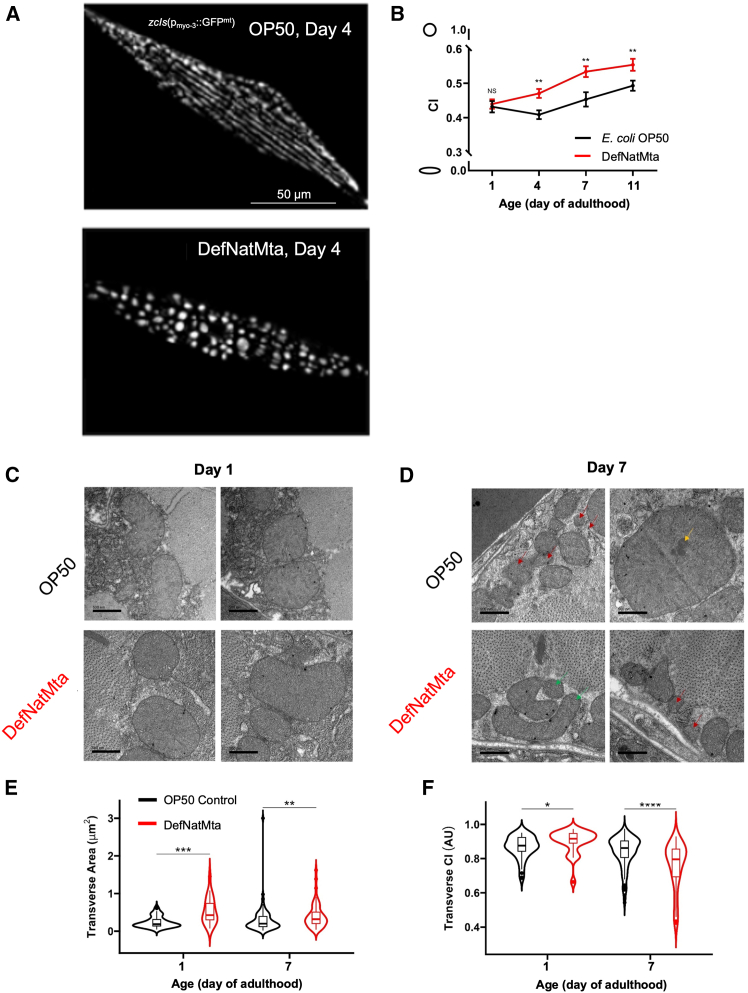
DefNatMta alters mitochondrial morphology and ultrastructure in body wall muscle (A) Representative images of mitochondrial morphology in body-wall muscle in *zcIs14*(*myo-3::GFP*^*mt*^) animals. Tubular networks in OP50-fed (top) and fragmented networks in DefNatMta-fed (bottom) animals at day 4 of adulthood. (B) DefNatMta-fed animals exhibit higher mitochondrial circularity on days 4, 7, and 11 of adulthood. *n* = 105 for DefNatMta, *n* = 107 for OP50; pooled from three biological replicates. Data are presented as mean ± SEM and analyzed via two-way ANOVA with post hoc FDR-corrected Student’s *t* tests. (C and D) Representative micrographs of mitochondria from transverse sections of the body wall muscle of OP50- and DefNatMta-fed worms during day 1 (C) and day 7 (D) of adulthood. Two representative images per condition are shown. Mitochondria with clear outer membrane disorganization (red arrows), electron-dense inclusions (yellow arrows), and elongated shape (green arrows) are highlighted. Scale bars, 500 nm. (E and F) Mitochondrial area (E) and mitochondrial circularity (CI; F) calculated from transverse sections represented in C and D. Mitochondrial ultrastructure and morphology was measured in all observable mitochondria per transverse section. n = 182 for DefNatMta, n = 83 for OP50; pooled from two sections. Data are presented as violin plots showing the distribution of the data. The central line represents the median, and the width of the plot reflects the density of the data points. Statistical analysis performed using Kruskal-Wallis tests with post-hoc FDR corrected Wilcoxon rank-sum tests. ∗∗∗∗*p <* 0.0001; ∗∗*p <* 0.01; ∗*p <* 0.05; ns, not significant (*p >* 0.05).

The lack of association between DefNatMta and changes in host body wall musculature and neuromuscular transmission led us to examine if DefNatMta mediates effects on age-related muscle function by altering mitochondria. The efficiency of mitochondrial networks is crucial for cellular bioenergetics and, consequently, for the efficiency of the *C. elegans* body wall muscles. Moreover, age-related changes in mitochondrial morphology have been suggested to contribute to mitochondrial dysfunction and progressive loss of muscle function.^44^^,^^45^ We imaged mitochondrial networks by using GFP-tagged mitochondria in body wall muscle^46^ and observed increasing mitochondrial circularity, a sign of mitochondrial fragmentation, with age in all animals (Figures 3A and 3B) regardless of bacterial source (ANOVA, age × bacterial source interaction: F_3, 203_ = 2.07, *p* = 0.1054; main effect of age: F_3, 203_ = 15.99, *p* < 0.0001). These findings are consistent with studies describing age-related alterations of mitochondrial morphology across species, including in muscle mitochondrial networks in *C. elegans*.^45^ Surprisingly, mitochondrial circularity was significantly increased in DefNatMta-fed animals (ANOVA, main effect of bacterial source: F_1, 203_ = 22.76, *p* < 0.0001) during days 4, 7, and 11 of adulthood (Figures 3A and 3B). This was unexpected, as fragmented mitochondrial networks are generally less bioenergetically efficient than fused networks,^47^ and yet, DefNatMta-fed worms displayed more circular mitochondria while maintaining swimming speeds equal to or greater than those of day 1 adult worms and OP50-fed controls. This led us to further examine mitochondrial structure using electron microscopy, which allowed us to assess both mitochondrial morphology and mitochondrial ultrastructure.

Animals in both conditions showed a uniform internal mitochondrial structure with fully intact and regular outer membrane morphologies and dense cristae in both conditions on day 1 of adulthood (Figure 3C). In day 7 adults, mitochondrial abnormalities consistent with pathology and aging were visible, including lack of a discernible outer membrane around all or part of the mitochondrion, sparse cristae, abnormally large mitochondria,^48^^,^^49^ and electron dense inclusions, which are small, dark structures visible under an electron microscope^44^ (Figure 3D). Mitochondria of day 1 adults fed on DefNatMta appeared larger and more circular than those of OP50-fed worms, consistent with a globular, fragmented mitochondrial network (Figure 3C). By day 7, mitochondria in DefNatMta-fed animals still appeared larger and also more elongated compared to age-matched OP50-fed worms (Figure 3D). The visually observed mitochondrial elongation in aged DefNatMta animals contradicts the results we obtained from GFP-tagged mitochondria, possibly as a result of differences in sample preparation (live imaging vs. chemical fixation) or orientation (transverse vs. longitudinal imaging) or related to imaging either GFP-tagged or wild-type mitochondria, but is consistent with the altered mitochondrial dynamics under different bacterial conditions. Together these results provide evidence that the DefNatMta influences host mitochondrial networks, altering mitochondrial size and circularity, without affecting mitochondrial ultrastructure.

### Effects of DefNatMta on motility requires DRP-1

**Figure 4:**
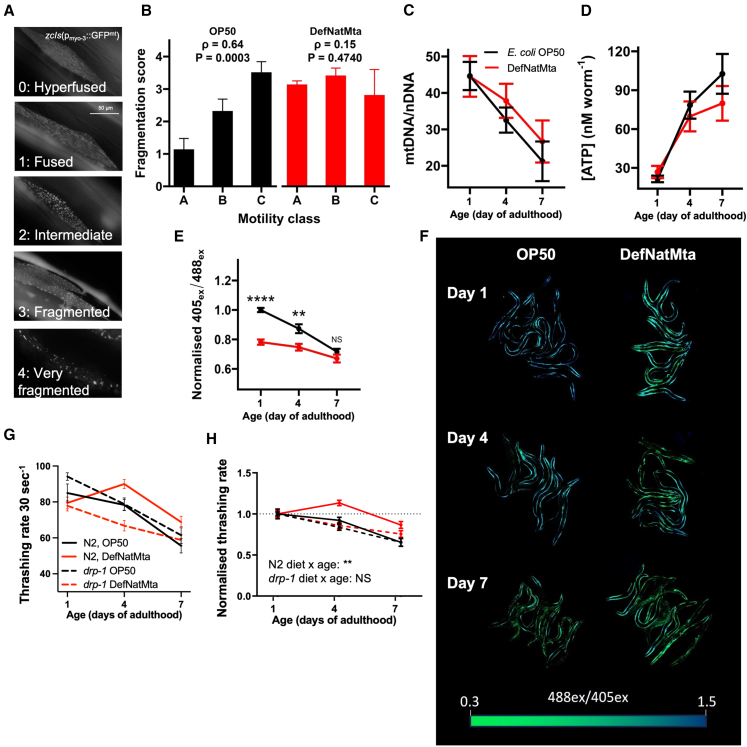
DefNatMta-associated effects on motility involve mitochondrial dynamics and require DRP-1 (A) Representative images of discrete numerical scores describing mitochondrial fragmentation in *zcIs14*(*myo-3::GFP*^*mt*^) animals. 0: Mitochondria are hyperfused; tubular networks of mitochondria are joined; 1, linear: tubular networks of mitochondria organized into linear tracts; 2, intermediate: both tubular and spherical mitochondria present in cell; 3, fragmented: mostly spherical mitochondria covering majority of cell; 4, very fragmented: large regions of cell devoid of GFP^mt^ signal. (B) Increased mitochondrial fragmentation scores are correlated with reduced motility in aged OP50-fed animals. The correlation is lost in DefNatMta-fed animals. Motility classes: A, highly mobile, spontaneous movement; B, mobile when prodded but no spontaneous movement; C, alive but not mobile. *n* = 26 for DefNatMta; *n* = 27 for OP50; pooled from two biological replicates. Data are presented as mean ± SEM and analyzed using Spearman’s rank-order correlation. (C) Whole-body mtDNA-CN measured in single animals in *glp-1* background by qPCR. Relative mtDNA-CNs were calculated by comparing the amplification of a mitochondrial gene (*nd-1*) and a nuclear gene (*cox-4*). *n* = 37 for DefNatMta, *n* = 34 for OP50; pooled from three biological replicates. Data are presented as mean ± SEM and analyzed via two-way ANOVA with post hoc FDR-corrected Student’s *t* tests. (D) Whole-body ATP levels quantified using a luminescence assay and a standard curve of ATP standards. *n* = 58 for DefNatMta, *n* = 56 for OP50; pooled from three biological replicates. Data are presented as mean ± SEM and analyzed via two-way ANOVA with post hoc FDR-corrected Student’s *t* tests. (E) 405_*ex*_/488_*ex*_ ratios calculated from raw fluorescence intensities of whole animals as represented in (F). n = 117 for DefNatMta, *n* = 121 for OP50; pooled from three biological replicates. Data are expressed as a proportion of the 405_*ex*_/488_*ex*_ ratios of day 1 adult OP50-fed animals and presented as mean ± SEM. Data were analyzed via two-way ANOVA with post hoc FDR-corrected Student’s *t* tests. (F) Representative full-body scans of animals expressing a Queen-2m ratiometric ATP sensor in the body wall muscles. Images are saturated for visualization. (G and H) Raw (G) and normalized thrashing rates (relative to day 1 adults; H) of OP50- and DefNatMta-fed worms with loss-of-function mutation in *drp-1*(*tm1108*) mutants. Thrashing rates were calculated manually. *n* = 239 for DefNatMta, *n* = 250 for OP50; pooled from two biological replicates. Data are presented as mean ± SEM and analyzed via two-way ANOVA. ∗∗∗∗*p <* 0.0001; ∗∗*p <* 0.01; ns, not significant (*p >* 0.05).

Given that DefNatMta both alters mitochondrial networks and motility, we explored if these effects are linked. We first examined if there was a correlation between mitochondrial fragmentation and motility. Animals with GFP-tagged muscle mitochondria were aged and classified according to their locomotory class (A: highly mobile, spontaneous sinusoidal locomotion; B: mobile only when prodded; C: alive but immobile even when prodded). Each animal was then imaged individually, and mitochondrial fragmentation severity was scored (Figure 4A). This allowed us to determine the motility class and mitochondrial fragmentation score of each individual. As expected, mitochondrial fragmentation scores were positively correlated with impaired motility in OP50-fed animals (Figure 4B; Spearman’s ρ = 0.64, *p* = 0.0004). In contrast, no correlation between motility class and mitochondrial fragmentation score was found in DefNatMta-fed worms (Spearman’s ρ = 0.14, *p* = 0.5110), suggesting DefNatMta uncouples mitochondrial network organization from age-related muscle function.

Mitochondrial fusion increases ATP synthesis, while fission enables the elimination of old or damaged mitochondria.^47^ Since our data indicated that DefNatMta alters mitochondrial network dynamics, we asked if DefNatMta affects mitochondrial health. We measured whole-body relative mtDNA copy number (mtDNA-CN) and ATP levels as indicators of mitochondrial health.^50^ mtDNA-CN is tightly linked to reproduction, doubling during the L4-adult moult.^51^ Since we had observed reproductive differences between OP50- and DefNatMta-fed animals, we performed all mtDNA-CN experiments in germline-free *glp-1*(*e2141*) animals to perform somatic measurements without confounding effects from germline or progeny mtDNA-CN. Relative mtDNA-CN was measured using mitochondrial DNA and nuclear DNA primer pairs (Table 1), and mtDNA-CN levels decreased sharply with age in both OP50- and DefNatMta-fed animals (ANOVA, main effect of age: F_2, 65_ = 9.30, *p* = 0.0002), but was not affected by bacterial source (Figure 4C; ANOVA, age × bacterial source interaction: F_2, 65_ = 0.22, *p* = 0.8041; main effect of bacterial source: F_1, 65_ = 0.45, *p* = 0.5036). Whole-body ATP levels increased with age in both OP50- and DefNatMta-fed animals (Figure 4D; ANOVA, main effect of age: F_2, 108_ = 22.62, *p* < 0.0001) but were similarly unaffected by bacterial source (ANOVA, age × bacterial source interaction: F_2, 108_ = 0.94, *p* = 0.3954; main effect of bacterial source: F_1, 108_ = 0.25, *p* = 0.6216). The observed increases in ATP levels with age are likely a result of age-related increases in body mass in *C. elegans*, which can have significant effects on bulk ATP levels.^53^

**Table 1:** Descriptions of mitochondrial and nuclear primer pairs

Target	Forward primer	Reverse primer	Amplicon size	Annealing temperature	Source
*nd-1* (mtDNA)	AGC GTC ATT TAT TGG GAA GAA GAC GCC	AAG CTT GTG CTA ATC CCA TAA ATG T	75 bp	62°C	Rooney et al.^50^^,^^52^
*cox-4* (nDNA)	GCC GAC TGG AAG AAC TTG TC	GCG GAG ATC ACC TTC CAG TA	164 bp	62°C	Rooney et al.^50^

While this indicated that no mitochondrial functional alterations could be detected at the whole-body level between DefNatMta- and OP50-fed animals, it did not exclude alterations within the body wall muscle. We therefore examined ATP levels specifically in body wall muscle using the ratiometric ATP sensor Queen-2m under the control of the *myo-3* promoter.^54^^,^^55^ The effects of DefNatMta on muscle ATP levels mirrored the effects we observed on swimming: DefNatMta-fed worms had significantly lower ATP levels relative to OP50-fed worms in early adulthood but exhibited less age-related decline (Figures 4E and 4F; ANOVA, age × bacterial source interaction: F_2, 232_ = 7.02, *p* = 0.0011) and had similar ATP levels to OP50-fed worms by day 7 of adulthood. These findings indicate that DefNatMta alters muscle bioenergetics specifically, without affecting whole-body ATP or mtDNA levels.

To better understand the mechanisms by which DefNatMta affects muscle function, we directly tested if mitochondrial network dynamics are involved in the motility phenotype utilizing mutants for *drp-1*, which encodes DRP-1. *drp-1* is required for mitochondrial fission, and *drp-1* mutants exhibit hyperfused mitochondrial networks.^56^ Forcing fusion of the mitochondrial networks using the *drp-1* mutant would allow us to specifically test whether fission is required for DefNatMta to affect motility. Thrashing assays in wild-type animals once again showed that age-related motility rates were improved by the DefNatMta (ANOVA, age × bacterial source interaction: F_2, 244_ = 4.97, *p* = 0.0076), whereas *drp-1* mutants showed similar rates of age-related motility decline regardless of bacterial source (Figures 4G and 4H; ANOVA, age × bacterial source interaction: F_2, 233_ = 2.25, *p* = 0.1078). These findings suggest that DefNatMta alters mitochondrial networks and bioenergetics in muscle, consistent with mitochondrial fission in the motility-preserving effects of DefNatMta.

## Discussion

The gut microbiota is complex, making experimental manipulation and demonstrating causation challenging. Using the genetically tractable model organism *C. elegans* combined with a microbial community derived from the natural *C. elegans* microbiota, DefNatMta, allows us to perform mechanistic studies in a more natural setting. We find that in comparison to the standard laboratory bacterial source *E. coli* OP50, a domesticated bacteria that does not colonize *C. elegans*, DefNatMta forms a stable gut microbial community. The formation of this gut microbiota is characterized by the enrichment of *Ochrobactrum* MYb71 and *Stenotrophomonas* MYb57, consistent with previous studies.^15^^,^^19^ DefNatMta contains several genera featured in a recently developed *C. elegans* microbiota resource, CeMbio.^57^ Similarly to CeMbio, DefNatMta enables more realistic studies whereby natural host-microbiota interactions are considered.

Our study reveals interactions by which a bacterial community alters muscle mitochondrial networks and bioenergetics, and motor function in the host. DefNatMta reduces motility in young adults while protecting against decline in motility later in life. The negative impact in early adulthood may reflect mild physiological stress, consistent with the effects of DefNatMta on reproduction. In contrast, the suppression of age-related decline suggests potential effects on muscle bioenergetics or proteostasis, consistent with the effects of DefNatMta on proteotoxicity. These findings imply that DefNatMta might exert modest early costs but confer long-term resilience to age-related decline in motility.

We found that DefNatMta impacts muscle ATP levels, mitochondrial networks, and motor function and protects against proteotoxicity in muscle. DefNatMta brings health benefits, while also reducing lifespan, raising the question if the phenotypes are linked or arise independently. The preservation of motility may result from processes that benefit tissue function without necessarily extending lifespan, such as altered proteostasis, stress resistance, or mitochondrial function, caused by microbial signaling. The shortened lifespan observed in DefNatMta-fed animals could reflect other factors, such as microbial pathogenicity. Alternatively, the effects on motility and lifespan are causally linked, with microbial signals or trade-offs in energy allocation underlying both phenotypes.

Mitochondria are essential components of eukaryotic cells, carrying out critical physiological processes that include metabolism and energy production. Mitochondria exist not only as isolated organelles but also as highly interconnected networks, shifting between these states via opposing fusion and fission forces. This ability to transition between fission/fusion states is essential for the functions of mitochondria in maintaining optimal cellular bioenergetics and in cellular stress responses.^44^ We showed that DefNatMta requires the regulator of mitochondrial fission *drp-1* to protect against age-related motility decline, consistent with regulation of mitochondrial fission by DefNatMta. Experiments testing additional mitochondrial network mutants as well as genetic rescues could provide more direct evidence that mitochondrial fission is involved. Our data do not support effects on motor function through changes in muscle structure. Instead, our findings suggest that DefNatMta influences motor function by disrupting mitochondrial networks, possibly as a consequence of metabolic changes, leading to altered ATP production in muscle. A possibility not explored here is microbial effects on the mitochondrial unfolded protein response, to refold or degrade misfolded proteins within the mitochondria, preserving muscle function and preventing damage.^58^ Additionally, future studies employing functional assays such as respirometry analysis would further demonstrate involvement of mitochondrial function.

Our study highlights mechanisms by which the gut microbiota influences muscle health during aging. Studies in humans show links between diet, microbiota composition, muscle function, and frailty, suggesting that targeting the microbiota through dietary or other interventions could improve health during aging.^59^^,^^60^ Studies using germ-free and antibiotic-treated mice have provided experimental evidence that the gut microbiota regulates skeletal muscle mass and function.^61^^,^^62^ Indoles produced by *E. coli* K12 increase age-related motility in *C. elegans, Drosophila*, and mice, suggesting that mechanisms by which the microbiota impact host lifelong health are conserved across species.^63^ In *C. elegans*, the polysaccharide colanic acid produced by *E. coli* regulates mitochondrial dynamics, protects against proteotoxicity derived from human Aβ, and extends lifespan, effects that are partially conserved in *Drosophila* and mice.^64^ Our study supports and adds to these findings by showing that the microbiota alters mitochondrial networks and metabolism in muscle, thereby providing mechanistic insight into the microbiota-muscle axis. We anticipate that future studies will add more mechanistic detail, e.g., if the gut microbiota alters key metabolic processes that contribute to mitochondrial and muscle function.^65^ Future work may also disentangle nutritional and colonization-dependent effects, e.g., by using heat-inactivated bacteria.

### Limitations of the study

DefNatMta represents a subset of the native microbiota and may not capture its full ecological complexity. Our focus was on motility and muscle mitochondria, leaving potential effects on other tissues unexplored. We did not directly measure mitochondrial respiration, and nutritional- vs. colonization-specific contributions were not fully disentangled. Finally, all experiments were conducted under laboratory conditions, which may not reflect natural environments.

## Resource availability

### Lead contact

Further information and requests for resources should be directed to and will be fulfilled by the lead contact, Marina Ezcurra (m.ezcurra@kent.ac.uk).

### Materials availability

The study did not generate new unique reagents.

### Data and code availability

• The raw data (e.g., measurements stored in Excel files, images) that support the findings of this study are available from the lead contact upon request.
• This article does not report any original code.
• No additional resources are reported.

## STAR★Methods

### Key resources table

**Table:** 

REAGENT or RESOURCE	SOURCE	IDENTIFIER
**Bacterial and virus strains**		

*Escherichia coli* OP50	Caenorhabditis Genetics Center	OP50
*Achromobacter* sp. F32 MYb9	Dirksen et al.^15^	MYb9
*Acinetobacter* sp. LB BR12338 MYb10	Dirksen et al.^15^	MYb10
*Pseudomonas lurida* MYb11	Dirksen et al.^15^	MYb11
*Arthrobacter aurescens* MYb27	Dirksen et al.^15^	MYb27
*Microbacterium oxydans* MYb45	Dirksen et al.^15^	MYb45
*Bacillus* sp. SG20 MYb56	Dirksen et al.^15^	MYb56
*Stenotrophomonas* sp. R-41388 MYb57	Dirksen et al.^15^	MYb57
*Ochrobactrum* sp. R-26465 MYb71	Dirksen et al.^15^	MYb71
*Leuconostoc pseudomesenteroides* MYb83	Dirksen et al.^15^	MYb83
*Chryseobacterium* sp. CHNTR56 MYb120	Dirksen et al.^15^	MYb120
*Pseudomonas tuomuerensis* MYb218	Dirksen et al.^15^	MYb218

**Chemicals, peptides, and recombinant proteins**		

Agarose	Melford	Cat# A20090–500.0 CAS 9012-36-6
Calcium chloride dihydrate	Sigma Aldrich	Cat# C5080-500G CAS 10035-04-8
Cholesterol	Sigma Aldrich	Cat# C8667-5G CAS 57-88-5
Dipotassium hydrogen phosphate	Supelco	Cat# 1.05104.1000 CAS 7758-11-4
Erioglaucine disodium salt	Sigma Alrich	Cat# 861146-5G CAS 3844-45-9
Ethylenediaminetetraacetic acid disodium salt dihydrate (EDTA)	Thermo Fisher	Cat# D/0700/53 CAS 6381-92-6
Glutaraldehyde	Sigma Aldrich	Cat# G7776 CAS 111-30-8
Lead(II) nitrate	Sigma Aldrich	Cat# 228621-100G CAS 10099-74-8
Levamisole hydrochloride	Supelco	Cat# PHR1798-250 MG CAS 16595-80-5
Magnesium sulfate	Melford	Cat# M24300–1000.0 CAS 7487-88-9
Nonidet P40 (NP40) substitute	Affymetrix	Cat# 19628 CAS 9002-93-1
Osmium tetroxide	Sigma Aldrich	Cat# 201030 CAS 20816-12-0
Potassium dihydrogen phosphate	Thermo Fisher	Cat# 011594.A1 CAS 7778-77-0
Propylene oxide	Sigma Aldrich	Cat# 110205-500 ML CAS 75-56-9
Proteinase K	New England Biolabs	Cat# P8102S CAS 39450-01-6
Sodium cacodylate trihydrate	Sigma Aldrich	Cat# C4945 CAS 6131-99-3
Sodium chloride	SLS	Cat# CHE3320 CAS 7647-14-5
Sodium citrate dihydrate	Sigma Aldrich	Cat# 1064480500 CAS 6132-04-3
Sodium hydroxide	Sigma Aldrich	Cat# S8045-500G CAS 1310-73-2
Sodium hypochlorite	Thermo Fisher	Cat# L14709.AP CAS 7681-52-9
Sodium phosphate dibasic	Sigma Aldrich	Cat# S0876-1 KG CAS 7559-79-4
SYBR Green PCR Master Mix	Thermo Fisher	Cat# 4309155
Tetramisole hydrochloride	Sigma Aldrich	Cat# L9756-10G CAS 16596-80-5
TRIS Base	Melford	Cat# T60040 (B2005) CAS 77-86-1
Triton X-100	Sigma Aldrich	Cat# X100-500 ML CAS 9036-19-5
Tryptone	BD	Cat# 211705
Tween 20	Sigma Aldrich	Cat# P1379-250 ML CAS 9005-64-5
Uranyl acetate	Polysciences	Cat# 21447 CAS 6159-44-0
Yeast extract	BD	Cat# 212750 CAS 8013-01-2

**Critical commercial assays**		

Agar Low Viscosity (LV) Resin Kit	Agar Scientific	Cat# AGR1078
CellTiterGlo 3D Cell Viability Assay	Promega	Cat# G9681
NEBNext Ultra DNA Library Prep Kit	New England Biolabs	Cat# E7370L
QIAGEN DNeasy Blood and Tissue Kit	QIAGEN	Cat# 69504
QIAGEN Gel Extraction Kit	QIAGEN	Cat# 28704

**Experimental models: Organisms/strains**		

*C. elegans*: N2 CGCM (wildtype)	Caenorhabditis Genetics Center	N2
*C. elegans*: GA2001 *wuls305 [myo-3p::Queen-2m]*	Galimov et al.^54^; Yaginuma et al.^55^	GA2001
*C. elegans*: CB4037 *glp-1 (e2141)*	Caenorhabditis Genetics Center	CB4037
*C. elegans*: RW1596 *myo-3 (st386); StEx30 [myo-3p::GFP::myo-3 +rol-6 (su1006)*	Caenorhabditis Genetics Center	RW1596
*C. elegans*: WX8490 *YqIs100 [ced-1p::mCHERRY::ACT1]*	Zhang lab, National Institute of Biological Sciences, Beijing	WX8490
*C. elegans*: SJ4103 *zcls14 [myo-3p::GFP(mit)*	Caenorhabditis Genetics Center	CU6372
*C. elegans*: CU6372 *drp-1 (tm1108)*	Caenorhabditis Genetics Center	CU6372
*C. elegans*: GMC101 *dvIs100 [unc-54p::A-beta-1-42::unc-54 3′-UTR +mtl-2p::GFP]*	Caenorhabditis Genetics Center	GMC101

**Oligonucleotides**		

*nd-1* (forward): AGC GTC ATT TAT TGG GAA GAA GAC GCC	Rooney et al.,^50^ Bratic et al.^66^	N/A
*nd-1* (reverse): AAG CTT GTG CTA ATC CCA TAA ATG T	Rooney et al.^50^, Bratic et al.^66^	N/A
*cox-4* (forward): GCC GAC TGG AAG AAC TTG TC	Rooney et al.^50^	N/A
*cox-4* (reverse): GCG GAG ATC ACC TTC CAG TA	Rooney et al.^50^	N/A
16S rRNA 515F-806R (forward): GTG CCA GCM GCC GCG GTA A	Caporaso et al.^67^	N/A
16S rRNA 515F-806R (reverse): GGA CTA CHV GGG TWT CTA AT	Caporaso et al.^67^	N/A

**Software and algorithms**		

Fiji ImageJ v. 2.15.1	Schindelin et al.^68^	https://imagej.net/software/fiji/
FLASH v. 1.2.7	Magoč, and Salzberg^69^	https://github.com/ebiggers/flash
GraphPad Prism v. 10	GraphPad	https://www.graphpad.com/
QIIME v. 1.70	Caporaso et al.^67^	https://qiime.org/1.4.0/
QuantStudio Design and Analysis	Thermo Fisher	https://www.thermofisher.com/
R v. 4.3.3	The Comprehensive R Archive Network	https://cran.r-project.org/
UCHIME v. 4.2.40	Edgar et al.^70^	https://drive5.com/usearch/manual/uchime_algo.html
Uparse v. 7.0.1001	Edgar^71^	https://drive5.com/uparse/
Zen Blue v. 2.3.69.1000	Zeiss	https://www.zeiss.com/microscopy/

### Experimental model and study participant details

#### Bacterial culture and strains

Strains *Achromobacter* sp. F32 MYb9, *Acinetobacter* sp. LB BR12338 MYb10, *Pseudomonas lurida* MYb11, *Arthrobacter aurescens* MYb27, *Microbacterium oxydans* MYb45, *Bacillus* sp. SG20 MYb56, *Stenotrophomonas* sp. R-41388 MYb57, *Ochrobactrum* sp. R-26465 MYb71, *Leuconostoc pseudomesenteroides* MYb83, *Chryseobacterium* sp. CHNTR56 MYb120 and *Pseudomonas tuomuerensis* MYb218^15^ were grown individually in LB (10 g/L tryptone, 5 g/L yeast extract, 5 g/L NaCl) at 25°C for 72 h to reach stationary phase, before being pooled in equal volumes to form the DefNatMta community. *Escherichia coli* OP50 was grown at 37°C in a 180 RPM shaking incubator overnight. 200 μL aliquots of culture (*E. coli* OP50) or culture mixture (DefNatMta) were seeded onto 60 mm nematode growth media (NGM) plates and allowed to dry at room temperature for three days prior to use.

#### *C. elegans* culture and strains

*C. elegans* were maintained under standard conditions,^72^ at 20°C on Nematode Growth Medium (NGM) plates seeded with *E. coli* OP50. All experiments were conducted at 20°C unless otherwise stated. For sterilisation and synchronisation, *C. elegans* were treated with bleach (4M NaOH, 5% vol/vol sodium hypochlorite) and washed with M9 (3 g/L KH_2_PO_4_, 6 g/L Na_2_HPO_4_, 5 g/L NaCl, 0.12 g/L MgSO_4_), followed by aliquoting eggs onto experimental plates. The following strains were used: N2 (wildtype N2 male stock, N2 CGCM), GA2001 *wuls305 [myo-3p::Queen-2m]*, CB4037 *glp-1 (e2141)*, RW1596 *myo-3 (st386)* V*; StEx30 [myo-3p::GFP::myo-3 + rol-6 (su1006)]*, WX8490 *YqIs100 [ced-1p::mCHERRY::ACT1]*, SJ4103 *zcls14 [myo-3p::GFP(mit)],* CU6372 *drp-1 (tm1108)* and GMC101 *dvIs100 [unc-54p::A-beta-1-42::unc-54 3′-UTR + mtl-2p::GFP].* WX8490 was a gift from Jennifer Tullet (University of Kent); all remaining strains were sourced from the Caenorhabditis Genetics Center.

### Method details

#### Gut colonisation

Gut colonisation was assessed by counting the number of colony-forming units (CFUs) recoverable from nematode samples following surface sterilisation as described in Walker et al.^73^ For each assay, 50 animals were anesthetized in 25 mM tetramisole in M9, surface-sterilised with a bleach solution (2.5% sodium hypochlorite, 2M NaOH) for 5 min and washed three times with M9. The efficacy of surface sterilisation was checked by examining CFU counts recoverable from the supernatant immediately following the final wash (500 μL aliquots were spread on LB plates and incubated for 24 h at 25°C). The suspension was homogenised in 1% Triton X-100 in M9 using a pellet pestle. The resulting suspensions were then centrifuged at 13 000 x g for 10 min, resuspended in M9, diluted at 10^−1^, 10^−2^ and 10^−3^ in PBS, and spread onto LB plates in technical triplicate. CFUs were then counted by eye following either 24 h incubation at 37°C (*E. coli* OP50) or 72 h incubation at 25° C (DefNatMta), and normalised to the number of worms per sample as follows:

$$\text{CFUs}\;{\text{worm}}^{-1}=\frac{\text{CFUs}*\text{Dilution}\;\text{factor}}{\text{Number}\;\text{of}\;\text{worms}}$$

#### Microbiome composition analysis

##### Genomic DNA extraction

Genomic DNA from *C. elegans* guts was extracted from worm lysates based on a modified version of a previous protocol.^19^ Briefly, age-synchronised populations from were washed into Eppendorf tubes, pelleted by centrifugation at 2500 RPM for 30 s, and washed twice with M9. The animals were surface-sterilised (2.5% sodium hypochlorite, 2M NaOH) for 5 min, and washed a further three times with M9. The efficacy of surface sterilisation was checked by examining CFU counts recoverable from the supernatant immediately following the final wash (500 μL aliquots were spread on LB plates and incubated for 24 h at 25°C). DNA was extracted using a QIAGEN DNeasy Blood and Tissue Kit and subject to three freeze-thaw cycles at −80°C and 37°C to release intestinal bacterial cells into solution. DNA purity and yield were assessed using a NanoDrop UV/Vis spectrophotometer and a Qubit Fluorometer, respectively. Genomic DNA from bacterial lawns were extracted in tandem to gut extractions. Contents of three three-day-old lawns seeded on NGM plates were scraped into 2 mL M9 using an inoculating loop. DNA extraction and quantification followed the same protocol outlined above.

##### 16 S rRNA sequencing

Sequencing of genomic DNA was performed by Novogene UK Ltd. Bacterial diversity was examined by PCR amplification of the V4 region of 16S rRNA with barcoded primers (515F-806R).^67^ PCR amplicons were quantified with SYBR Green, pooled in equidensity ratios, and purified using a QIAGEN Gel Extraction Kit. Library preparation was performed using an NEBNext Ultra DNA Library Prep Kit, and sequencing with an Illumina paired-end platform with a read length of 250 bp. Paired-end reads were merged with FLASH v. 1.2.7.^69^ Quality filtering was conducted using QIIME v. 1.7.0^70^ with default settings. Chimeric sequences were removed using the UCHIME algorithm with the Gold reference database.^71^ Operational taxonomic units (OTUs) were constructed using Uparse v. 7.0.1001^74^ with a minimum percent identity of 97%. Species assignment of representative OTU sequences was conducted using the Ribosomal Database Project Classifier v. 2.2.^75^ OTU sequences failing to match published DefNatMta sequences were removed (<5% of all reads). OTU abundances were normalised using a standard sequence number corresponding to the sample with the fewest sequences.

##### Diversity analyses

All diversity analyses were performed with the *vegan* package for R v. 4.3.3 using normalised OTU abundances. Alpha diversity was assessed using the Shannon diversity index and Student’s t tests. Beta diversity was assessed via permutational multivariate analysis of variance (MANOVA, 999 permutations) of Bray-Curtis distances using the *adonis* function. Principal coordinates analysis (PCoA) of Bray-Curtis distances was conducted using the *capscale* function and the relationships between environmental variables (taxon abundance) and the ordination axes were assessed using the *envfit* function with 999 permutations. *envfit* computes the direction of the effects of continuous variables by calculating the direction of maximum correlation between a given continuous variable and the ordination scores. The statistical significances of these variables are then assessed using a permutation test.

#### Bacterial choice assay

Bacterial-choice behavior was assessed using a modified version of a previous protocol.^76^ Worms were collected in groups of at least 20, washed three times with M9, and transferred onto the centers of 30 mm NGM plates seeded with 15 μL lawns of *E. coli* OP50 and the DefNatMta mixture, with each lawn spaced 20 mm apart. The plates were then incubated at 20°C for 24 h and imaged with an MBF Biosciences WormLab Imaging System. The number of worms residing in each bacterial lawn were counted by eye and a bacterial preference index was calculated as follows:

$$\text{Bacterial}\;\text{Preference}\;\text{Index}=\frac{N\;\left(\text{DefNatMta}\right)-N\;\left(E.\;coli\;\text{OP}50\right)}{N\;\left(\text{Total}\right)}$$

With this index, a value of 1 occurs when all animals associate with the DefNatMta mixture, and a value of −1 occurs when all animals associate with *E. coli* OP50. The total number of animals tested was 705 for the DefNatMta mixture and 1164 for *E. coli* OP50.

#### Body length analysis

Wildtype nematodes were suspended individually in a single drop of M9 buffer on glass slides and imaged with an MBF Biosciences WormLab Imaging System. Images were processed using Fiji. Segmented lines were drawn from the tip of nose to the tapered tip of the tail through the midline of the nematode. Calibrations were consistently maintained across all images. 10 animals were used per condition and experiment.

#### Brood size

Brood size was assessed as described in Hodgkin and Barnes.^77^ Briefly, individual worms were transferred at the L4 stage to separate NGM plates. Subsequently, they were transferred each day, up to day 6 of adulthood, to freshly seeded NGM plates to lay eggs. The viable progeny hatching from each brood were then counted at the late larval to adult life stage. Twelve animals were used per condition and experiment.

#### Developmental rates

Developmental rates were assessed via light microscopy 60 h after age synchronisation. Nematodes were anesthetized in 25 mM tetramisole and mounted onto 2.5% agarose pads in groups of approximately 50. Each animal was observed using a Leica DMR compound epifluorescence microscope with a 40× objective and scored based on vulval structure and the presence or absence of oocytes as described in Mok et al.^78^ The scoring criteria were: L3 = No vulva apparent; Early L4 = From the initial appearance of the vulval lumen (L4.1) to the separation of the vulval and uterine lumens (L4.4); Late L4 = From the appearance of the concave curvature between vulval cells vulD and vul B2 (L4.5), to the closure of the vulval lumen (L4.9); Adult = Fertilised oocytes apparent.

#### Pharyngeal pumping

Nematodes were transferred individually to freshly seeded NGM plates and allowed to acclimatise for 1 min. Following acclimation, the animals were manually observed for 20 s using a Leica S6 E Stereo Zoom light microscope and the number of pharyngeal pumps counted.

#### Lifespan measurements

Lifespan assays were conducted manually on NGM plates, with 25–30 animals per plate, and transferring animals daily until cessation of reproduction. Any animals that died from internal hatching were censored. Four biological replicates were conducted with approximately 100 animals per condition.

#### Defecation rates

Defecation rates were assessed as described previously.^79^ Individual nematodes were transferred to fresh NGM plates and allowed to acclimatise for 5 min. Following acclimation and the first peristaltic contraction of the body wall muscle, the time between four defecation motor programmes (DMPs) was measured. If no such contraction was observed over a 5-min period, the animal was classified as a non-defecator (0 DMPs min^−1^). Observations were conducted by eye using a Leica S6 E Stereo Zoom light microscope. Only nematodes observed to be in the process of feeding were assayed.

#### Intestinal barrier function

Intestinal barrier function was assessed using a modified version of a previous protocol.^68^ On day 11 of adulthood animals were washed three times with S-buffer (100 mM NaCl, 6.5 mM K_2_HPO_4_ and 43.5 mM KH_2_PO_4_ and suspended in a liquid suspension (1:1) of buffer mixed with blue food dye (Erioglaucine disodium salt, Sigma-Aldrich, 5.0% wt/vol in water) for 3 h. Animals were then washed three times with S buffer, centrifuged at 3500 rpm for 1 min, anesthetized in 25 mM tetramisole, and mounted on 2.5% agarose pads. The worms were visually analyzed for the presence or the absence of blue dye in the body cavity using a Leica DMR compound epifluorescence microscope with a CellCam Rana 200CR color cameraWorms were scored as “smurfs” if they appeared blue in their body cavity. 20–25 animals were used per condition and experiment.

#### Exploration assay

A single animal was placed on a 60 mm NGM plate containing 5 mL NGM media and seeded with 200 μL bacterial culture. Animals were allowed to explore the plate for 4 h at 20°C. Subsequently, the animals were removed and their tracks were imaged using an MBF Biosciences Worm Lab Imaging System. The total length of each track was calculated per animal using the segmented line tool for Fiji v. 2.15.1.

#### Oscillatory rheology

Rheology studies were performed using Anton Paar MRC 302 modular compact rheometer with an upper geometry cylinder (cylinder-relative ST10-4V-8.8/97.5). Bacterial lawns were scraped off NGM plates to generate samples (1.0 mL volume) Samples were analyzed in glass vials (Fisherbrand type III lime glass specimen vials, diameter: 19 mm, volume: 8.0 mL). Each experiment was conducted in triplicate. The sample was positioned on the rheometer and set with a relaxation time of 5 min with the geometry present. Oscillatory amplitude experiments maintained a frequency of 10 rad s^−1^ and performed with the amplitude of oscillation from 0.01% up to 100% at 298 K. Oscillatory frequency sweep experiments maintained a constant shear strain (γ) of 0.05% with an increasing frequency from 0.1 to 100 rad s^−1^ at 298 K. Viscosity was measured at shear rates of 0.1/s and 100/s.

#### Motility assessment

Manual motility analysis was performed by transferring single worms into 100 μL droplets of M9, waiting for 30 s, and counting the number of thrashes (defined as a deviation of the head and tail from the long axis of the body) for next 30 s. Automated motility analysis was performed using the MBF Biosciences Worm Lab Imaging System. Nematodes were transferred into 50 μL droplets of M9 placed on the inside of a Petri dish lid, and 1-min videos were taken to assess their swimming dynamics. Thrashing rates were quantified by extracting the total number of body bends (>20° deviation from the long axis of the body) over the length of the footage. Manual and automated thrashing rate measurements were compared using a subset of the total captured footage and were found to be closely correlated (Pearson’s correlation: r = 0.99, *p* < 0.0001; *n* = 30 animals, data not shown). Alternative metrics of swimming behavior were calculated directly from the swimming statistics summary provided by the MBF Biosciences Worm Lab imaging software as described in Restif et al.^33^ 10 animals were used per condition and experiment.

#### Aβ-associated paralysis assay

The GMC101 *dvIs100 [unc-54p::A-beta-1-42::unc-54 3′-UTR + mtl-2p::GFP]* strain was used for this assay. This strain carries a temperature-activated transgene, allowing the human Aβ isoform Aβ1-42 to be expressed in body wall muscle following a shift from 20°C to 25°C. The proportion of paralyzed animals was recorded on days 1–4, at approximately the same time each day. Paralyzed nematodes were classified as being incapable of forward and backward movement when prodded but still alive i.e., moving nose or tail, feeding, laying eggs. 100 animals were used per condition and experiment.

#### Quantitative reverse transcription-PCR for the expression level of the Aβ gene

GMC101 *punc-54*::Aβ1-42:*unc-54* 3′-UTR; *pmtl-2*::GFP animals were used for this assay. Animals incubated at 25°C from L4 stage, were collected at day 1 of adulthood and washed in M9 buffer. The pellet was resuspended in 50 μL M9. After addition of 200 μL TRIzol (Invitrogen), six freeze thaw cycles were carried out before the supernatant was transferred to a separate Eppendorf tube. The pellet was freeze-thawed a further four times and ground between freezes. The samples were resuspended in 50 μL TRIzol and stored at −80°C until use. Total RNA was extracted using the Direct-zol RNA Mini Prep Kit (Zymogen), using the protocol provided. cDNA was synthesised from extracted RNA diluted to 200 ng/μL in nuclease free water (Promega) using the Ultrascript cDNA Synthesis Kit (PCRBIOSYSTEMS) as per the protocol provided. qPCR was performed using 2× SyGreen Mix Lo-ROX (PCR BIOSYSTEMS) and 500 nM of primers with QuantStudio3 (Applied Biosystems). Relative expression of amyloid-β was analyzed using the comparative cycle threshold method (2−ΔΔCt). Four biological replicates were analyzed each with three technical replicates in the qPCR step. Three technical replicates for each sample were used for the qPCR assay, and four biological replicates were analyzed. Primers used for Aβ were (Forward) 5′-CAGAATTCCGACATGACTCAGGATATGAAG-3′ and (Reverse) 5′-CCCACCATGAGTCCAATGATTGC-3' and for *cyc-1* (Forward) 5′-CTAAACGTGGATTGGCGGCC-3′ and (Reverse) 5′-GCCCATGGTAAAGCGTACGG-3'.

#### Epifluorescence microscopy

##### Sample preparation and imaging

Fluorescence imaging of the body wall muscles was performed using GFP-tagged myosin (RW1596 *myo-3 (st386); StEx30 [myo-3p::GFP::myo-3 + rol-6 (su1006)]*) and mCherry-tagged actin (WX8490 *YqIs100 [ced-1p::mCHERRY::ACT1]*). Nematodes were anesthetized in 25 mM tetramisole and mounted on 2.5% agarose pads for imaging using a Leica DMR compound epifluorescence microscope fitted with a 40× objective. All images of the body wall muscles were captured between the pharynx and vulva.

Fluorescence imaging the body wall muscle mitochondrial networks was performed using mitochondrially-targeted GFP under the control of the *myo-3* promoter (SJ4103 *zcls14 myo-3p::GFP[mit]*). Nematodes were anesthetized in 25 mM tetramisole and mounted on 2.5% agarose pads for imaging using a Leica DMR compound epifluorescence microscope fitted with a 63× objective. All images were captured approximately equidistant from the pharynx and vulva.

##### Quantitative measurements of body wall muscle cell area and fiber length

Quantitative measurements of body wall muscle were performed as previously described.^39^ Briefly, muscle:area ratio (gap to total cell area) was calculated from images that included at least on complete muscle cell. Gaps left by degenerating muscle fibers and the total area of a single muscle cell were drawn and calculated in Fiji using the polygon selection tool. 15–20 animals were used per condition and experiment.

##### Body wall muscle organisation

Sarcomere disorganisation was assessed qualitatively using a scoring system.^32^ Cells received a score of 1 if all filaments were arranged in parallel, symmetric rows; a score of 2 if the filaments were largely arranged in parallel, but contained some gaps; 3 if the filaments appeared crooked or frayed, with an elevated number of gaps between filaments; and 4 if the filaments appeared mostly crooked, broken, and poorly arranged, with substantial gaps between filaments. 15–20 animals were used per condition and experiment.

##### Mitochondrial network integrity

The integrity of the mitochondrial networks was assessed quantitatively and qualitatively. To quantify mitochondrial shape, a circularity index (CI) was calculated as previously described.^44^ Circularity is a function of area and perimeter:

$$\text{CI}=4\pi \frac{\text{area}}{{\text{perimeter}}^{2}}$$

which fits each object to a perfect circle and measures its deviation, where 1 = a perfect circle and 0 = a straight line.

Qualitative scoring of network integrity was performed as follows: cells received a score of 1 if the network was entirely tubular and linear; 2 if the network was mostly linear with few spherical mitochondria; 3 if the network appeared mostly fragmented with spherical mitochondria predominant; and 4 if the network was almost entirely fragmented with large regions devoid of observable mitochondria.

#### Electron microscopy

##### Sample preparation and imaging

Nematodes were individually transferred into small quantities of M9 using an eyelash pick, before being fixed in 2.5% glutaraldehyde fixative in 100 mM sodium cacodylate buffer (CAB; pH 7.2). The heads and tails were separated from the bodies using a scalpel, and bodies were left overnight in fixative at 4°C. The samples were subsequently washed twice with CAB and re-suspended in 2% low melting-point agarose in CAB. The worm bodies were identified in the agarose suspension using a Leica S6 E Stereo Zoom light microscope, excised, transferred to glass vials, and stained with 1% osmium tetroxide in CAB for 1 h at room temperature. Excess stain was removed by washing twice with Milli-Q water, each wash lasting 10 min. Subsequently, worms were dehydrated in an ethanol series (50%, 70%, 90%, and 100%) for 10 min each, with the 100% ethanol dehydration step repeated 3 times. Dehydration was followed by two 10-min washes in propylene oxide, after which the samples were treated with a 1:1 mixture of low-viscosity (LV) resin and propylene oxide for 30 min at room temperature. Samples were then transferred to fresh LV resin twice for 2 h each before being embedded in LV resin via polymerisation at 60°C for 24 h. Embedded samples were examined under a Leica S6 E Stereo Zoom light microscope to identify and excise the worm bodies, which were then oriented on a resin block for optimal sectioning. Transverse section of 70 nm thickness were cut, approximately equidistant from the vulva and the anterior tip of the worm body, using a Diatome diamond knife and an EM UC7 ultramicrotome. Sections were collected onto 400-mesh copper grids and counterstained with 4.5% uranyl acetate for 45 min, followed by Reynolds' lead citrate (80 mM lead(II) nitrate, 120 mM sodium citrate; pH 12) for 7 min. Sections were imaged using a Jeol 1230 transmission electron microscope with an accelerating voltage of 80 kV, fitted with a Gatan One View 4K digital camera.

##### Myosin filament density

Myosin filament density was estimated from electron micrographs by manually counting the number of thick filaments in 10 0.65 x 0.52 (length x width) μm cross sections per replicate, with each section spanning the center of the sarcomere. Cross sectioning was performed blind using the File Name Encrypter tool within the Blind Analysis tool suite for Fiji v. 2.15.1.^66^

##### Mitochondrial morphology and ultrastructure

Mitochondrial morphology was estimated from electron micrographs by manually segmenting all observable mitochondria using the segmented line tool for Fiji v. 2.15.1^66^ and Click or tap here to enter text.quantified using the circularity index described in Epifluorescence microscopy. Mitochondrial ultrastructure was assessed qualitatively.

#### Muscular contractibility

Muscular contractibility was assessed using the acetylcholine esterase inhibitor levamisole. Groups of 10 nematodes were transferred in technical triplicate to unseeded 30 mm NGM plates and immediately imaged using an MBF Biosciences Worm Lab Imaging System. Immediately after imaging, the animals were transferred to freshly prepared NGM plates containing levamisole (75 μM). Animals were imaged after 10 min, and body lengths were quantified in both sets of images using the segmented line tool for Fiji v. 2.15.1. Muscular contractibility was then calculated as the plate-averaged % reduction in body length following levamisole treatment:

$$\text{Contractibility}\;\left(\%\right)=100*\left(1-\frac{\text{Body}\;\text{length}\;\left(+\text{levamisole}\right)}{\text{Body}\;\text{length}\;\left(-\text{levamisole}\right)}\right)$$

#### Gentle touch assay

Responses to gentle touch sensitivity were assessed as previously described.^80^ An eyebrow pick was used to stroke nematodes in two locations: immediately posterior to the pharynx (to distinctly stimulate the anterior touch response) and immediately anterior to the anus (to distinctly stimulate the posterior touch response). Nematodes were determined to be touch responsive if they stopped moving toward the eyebrow or moved away from the eyebrow. Each nematode was tested five times anteriorly and five times posteriorly and the percentage of touches responded to (out of 10) was recorded.

#### Mitochondrial function assays

##### mtDNA copy number

Germ-free *glp-1* animals cultivated at 25°C were used to quantify mtDNA copy number (mtDNA-CN). mtDNA-CN was measured using an adapted one-worm quantitative PCR (qPCR) protocol.^50^ Individual worms in lysis buffer (30 mM Tris pH 8, 8 mM EDTA, 100 mM NaCl, 0.7% NP40, 0.7% Tween 20, 100 μg mL^−1^ Proteinase K) were centrifuged briefly, and lysed (60 min at 65°C, 15 min at 95°C). PCR reactions were assembled in technical duplicate with mtDNA and nDNA (nuclear DNA) primers (Table 1) using an SYBR Green PCR Master Mix (Thermo Fisher), with 2 μL template DNA and a total reaction volume of 25 μL. PCR reactions were cycled in a QuantStudio 3 Real-Time PCR System (2 min at 50°, 10 min at 95°C, 40 cycles of 15 s at 95°C, 60 s at 62°C). Relative mtDNA-CNs were calculated using mitochondrial and nuclear-target cycle times (CTs) obtained from the QuantStudio Design and Analysis software, where:

$$\text{mtDNA}-\text{CN}=2^{{\;\text{nDNA}}_{\text{CT}}-{\text{mtDNA}}_{\text{CT}}}$$

Samples were excluded from copy number calculations if the CT standard deviation exceeded 1 for either nDNA or mtDNA primer pair within each technical replicate group.

##### Whole body ATP measurements

Whole-body ATP levels were assessed as described in Galimov et al.^54^ Briefly, worms were transferred in groups of five to 50 μL M9 in thin-walled PCR tubes. The samples were then lysed at 95 ^O^C for 15 min, incubated at 25 ^O^C for 5 min, and assessed using a luminescence-based CellTiterGlo 3D Cell Viability Assay (Promega) in accordance with the manufacturer’s instructions. ATP levels were quantified using a standard curve of ATP standards. Luminescence measurements were taken using a BMG FLUOStar Omega plate reader.

##### ATP measurements in body wall muscle using Queen-2M

For estimates of relative ATP levels using the Queen-2m sensor, a dual λ_ex_ of 400 and 488, and a λ_em_ of 545 were measured using a Zeiss LSM880confocal laser scanning microscope with a 40× objectiveClick or tap here to enter text. Images were captured as tile-scanned z-stacks through the entire range of observable fluorescence (40–70 μm) and stitched automatically with Zen Blue (Zeiss). The number of slices per stack were determined using the optimal sectioning tool of Zen Blue, with a 50% overlap between slices. The resulting scans were maximally projected for image analysis, and relative ATP levels were calculated by dividing the mean whole-body fluorescence at 405 nm λ_ex_ by the mean whole-body fluorescence at 488 nm λ_ex_ as described in Galimov et al.,^54^ and Yaginuma et al.^55^ These ratios were then expressed as a proportion of the 405ex/488ex ratios of day 1 adult *E. coli* OP50-fed controls.

### Quantification and statistical analysis

Statistical analysis was performed using GraphPad Prism 10 and R v. 4.3.3. Analysis of 16S rRNA data was performed as described in Microbiome composition analysis. Food preference data were analyzed using a Student’s *t* test. Developmental rate data were analyzed using a Wilcoxon rank-sum test. Survival data were analyzed using a log rank (Mantel-Cox) test. Intestinal permeability data were analyzed using a Fisher’s exact test. Rheology data were analyzed via two-way ANOVA with post-hoc Fisher’s LSD tests. Sarcomere organisation scores were analyzed via ordinal logistic regression with the package *ordinal* for R v. 4.3.3; to assess the significance of bacterial source, age and their interaction, we compared two cumulative link models using a likelihood ratio test: a null model, including only an intercept, and a full model, including bacterial source, age and their interaction as predictors. Post-hoc testing of sarcomere scores was performed using Wilcoxon rank-sum tests. Mitochondrial size and shape data from electron microscopy experiments were analyzed using Kruskal-Wallis tests with post-hoc Wilcoxon rank-sum tests. All remaining age-related data were analyzed via two-way ANOVA, with age and bacterial source included as an interaction term, and post-hoc testing was conducted using Student’s t tests. For all post-hoc comparisons, multiple comparisons were corrected using the False Discovery Rate (FDR).

## Acknowledgments

We acknowledge support from the Biotechnology and Biological Sciences Research Council (BBSRC) through grant BBSRC (BB/V011243/1) awarded to M.E., a BBSRC SoCoBio studentship awarded to L.M.F., and a Jane Irons PhD Studentship from Dr. John Stolz awarded to N.D., L.-J.W., and J.R.H. to thank the 10.13039/100014013UKRI (MR/Y03385X/1) for funding. Some strains were provided by the CGC, which is funded by the NIH 10.13039/100016958Office of Research Infrastructure Programs (P40 OD010440). We thank Jennifer Tullet (10.13039/501100001316University of Kent) and Nazif Alic (10.13039/501100000765University College London) for meaningful discussions on the manuscript.

## Author contributions

N.D., M.V.-P., L.M.F., and F.X. conducted the main bulk of experiments and analyses; A.A.K. carried out thrashing experiments; W.G.S. carried out and analyzed roaming experiments; B.K. carried out fluorescence imaging experiments and analyses; I.B. performed the TEM imaging; L.-J.W. conducted and analyzed the rheology experiments, J.R.H. supervised the rheology experiments, N.D., M.V.-P., and M.E. wrote the manuscript; and M.E. supervised the work and acquired funding.

## Declaration of interests

The authors declare no competing interests.
