# Supplementary material for: A microbial community that alters mitochondrial morphology and age-related motor function in C. elegans
Source: iScience. 2025 Nov 19;28(12):114128. doi: 10.1016/j.isci.2025.114128 (PMC12721190; doi:10.1016/j.isci.2025.114128)
Supplement: Document S1. Figures S1–S3 [file mmc1.pdf]

iScience, Volume *28*

Supplemental information

A microbial community that alters mitochondrial morphology and age-related motor function in *C. elegans*

Nathan Dennis, Mireya Vazquez-Prada, Laura M. Freeman, Feng Xue, Lisa-Jane White, Antonis A. Karamalegos, William G. Sullivan, Brigita Kudzminkaite, Ian Brown, Jennifer R. Hiscock, and Marina Ezcurra

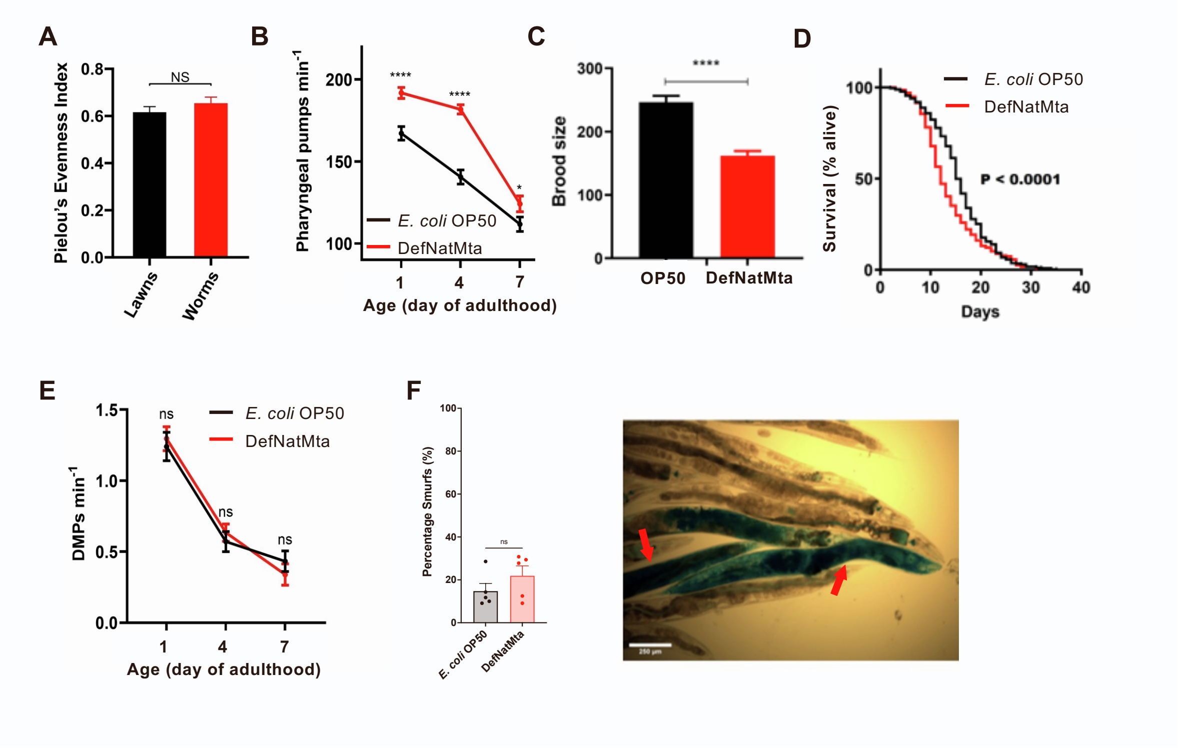

Figure S1.

A) Pielou's diversity indices of DefNatMta lawns and DefNatMta-fed animals. N = 5 biological replicates, data are presented as mean ± SEM and analysed via Student's t test. **B**) Pharyngeal pumping rate of DefNatMta and *E. coli* OP50-fed worms. n = 150 per condition; pooled from four biological replicates. Data are presented as mean ± SEM and analysed via two-way ANOVA with post-hoc FDR-corrected Student's t tests. **C**) Total brood size of DefNatMta and *E. coli* OP50-fed worms. n = 36 per condition; pooled from three biological replicates. Data are presented as mean ± SEM and analysed via Student's t test. **D**) Survival rate of DefNatMta and *E. coli* OP50-fed worms. n = 476 for OP50, n = 419 for DefNatMta, pooled from four biological replicates (left panel). Median lifespan for each replicate (right panel). Data are analysed using Log Rank (Mantel-Cox) Test. **E**) Quantification of defecation rate; defecation motor program (DMP). n = 90 per condition; pooled from three biological replicates. Data are presented as mean ± SEM and analysed via two-way ANOVA with post-hoc FDR-corrected Student's t tests. **F**) Quantification of intestinal barrier function. Bars represent the grand mean ± SEM. Each dot represents the mean value from a single trial (left). Representative image (right). 'Smurf' refers to animal with intestinal leakage of blue dye into body cavity. Red arrows point to animals with a 'Smurf' phenotype. n = 78 for DefNatMta, n = 114 for OP50; pooled from five biological replicates. Data are analysed using Fisher's exact test. ****, P < 0.0001; **, P < 0.01*; P < 0.05; ns, not significant (P > 0.05).

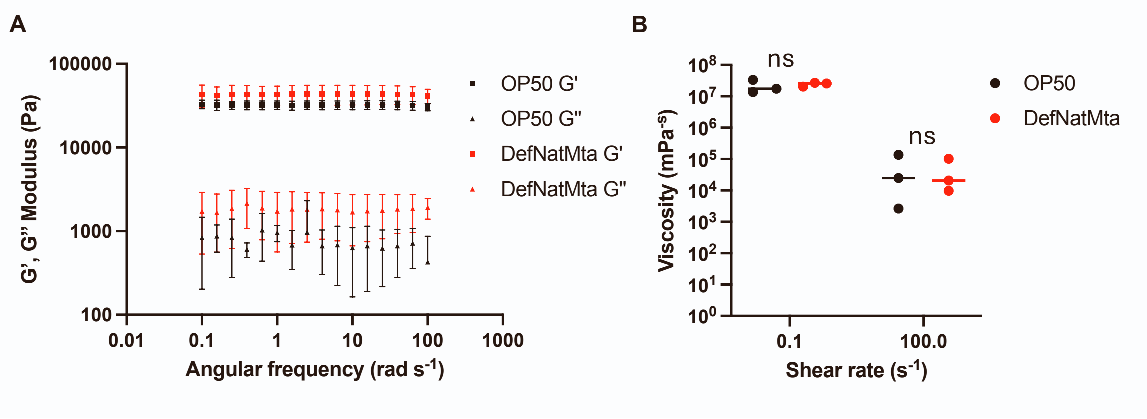

Figure S2. Rheology measurements of OP50 and DefNatMta bacterial lawns.

A) Frequency sweep measurements at 298 K (25ºC) for OP50 and DefNatMta, obtained from the linear viscoelastic region under a constant shear strain (γ) of 0.05 %. The linear viscoelastic region (LVR) for these bacterial lawns were identified via amplitude sweep experiments (frequency = 10 rad s^{-1} from 0.01 % up to 100 % at 298 K). Bars represent the mean ± SD. **B**) Viscosity recorded at shear rates of 0.1 and 100/s. The lower viscosity measures at 100/s are suggestive of the fluid within the lawn to flowing more freely due to disruption of the material structure. Flat lines represent the mean. Each dot represents the value from a single trial. All rheological data were measured in triplicate at 298 K. Data are analysed using two-way ANOVA with Fisher's LSD test. ns, not significant ($P > 0.05$).

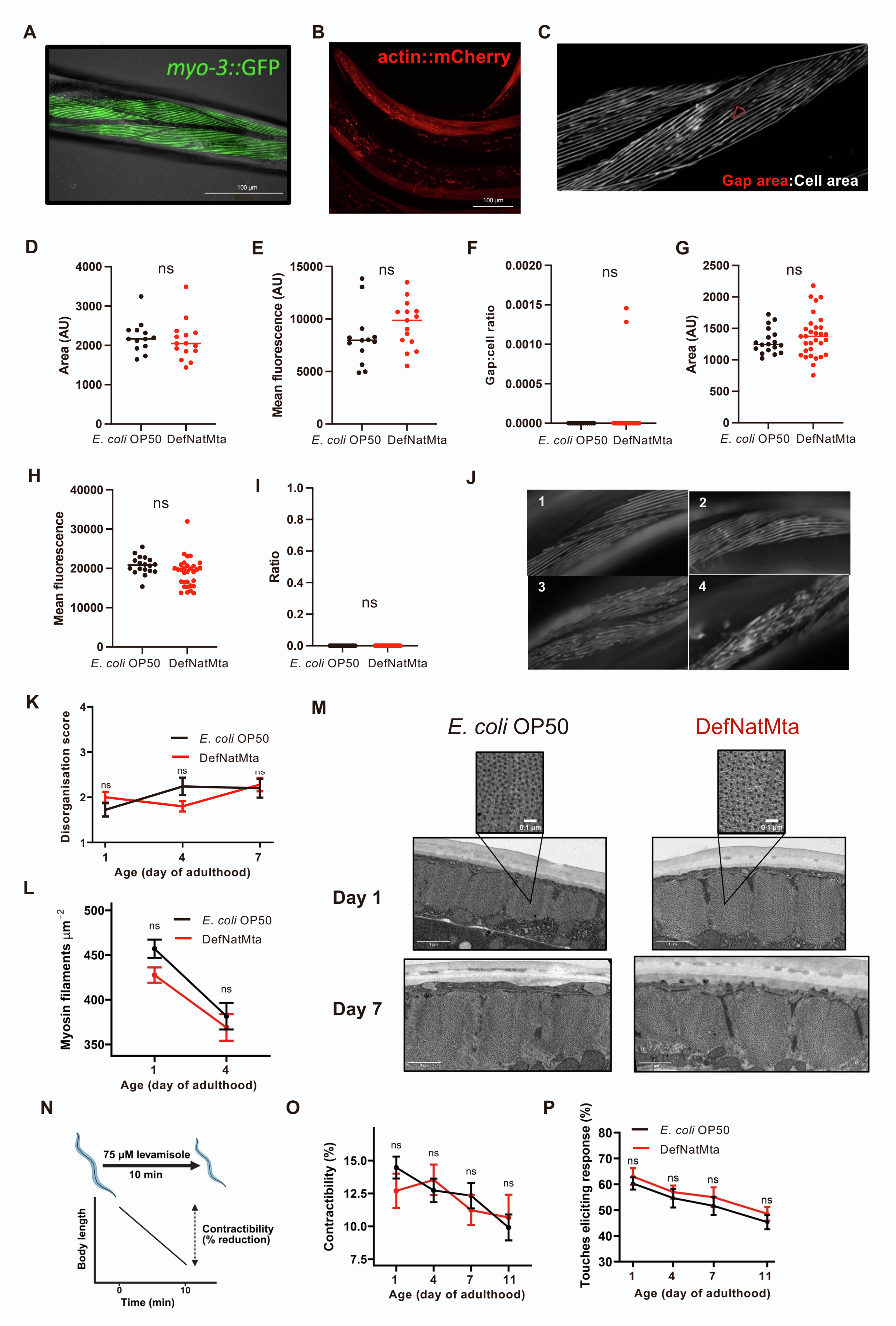

Figure S3.

A) Representative image of muscle cells expressing *myo-3::GFP* (RW1596 *myo-3 (st386); StEx30[myo-3p::GFP::myo-3 + rol-6 (su1006)]*), showing the anterior half of the worm. **B**)

Representative image of muscle cells expressing *ced-1::mCherry* (WX8490 *YqIs100 [ced-1p::mCherry::ACT1]*). **C**) Example image of how the muscle area ratio (gap to total cell area) was calculated. Gaps left by degenerating muscle fibres (red-lined area) and the total area of a single muscle cell (white-lined area) were drawn and calculated in Fiji using the polygon selection tool. **D-F**) Measures of GFP-tagged myosin in single muscle cells of day 1 animals. Area covered (D), mean fluorescence (E), Gap:cell area ratio (F). n = 12-16 per condition; pooled from two biological replicates. Data are presented as values for individual animals and mean. Statistical analysis performed using Student's t test. **G-I**) Measures mCherry-tagged actin in single muscle cells. Area covered (G), mean fluorescence (H), Gap:cell area ratio (I). n = 12-27 per condition; pooled from two biological replicates. Data are presented as values for individual animals and mean. Statistical analysis performed using Student's t test. **J**) Representative images of scoring system for assessing sarcomere disorganisation. 1 = Fibres organised in parallel, symmetric rows; 2 = Fibres mainly parallel, containing some gaps; 3= Fibres lie in same direction but contain gaps, bends, are frayed; 4 = Fibres are broken and bent with a 'blurred' appearance. **K**) Sarcomere disorganisation scores. n = 81 for DefNatMta, n = 102 for OP50; pooled from three biological replicates. Data are presented as mean ± SEM. Data were analysed via ordinal logistic regression with post-hoc FDR-corrected Wilcoxon rank-sum tests. **L**) Myosin filament densities, assessed from cross-sections (as in M insets) of randomly chosen muscle blocks. n = 20 per condition; pooled from two replicates, data are presented as mean ± SEM and analysed using two-way ANOVA with post-hoc FDR-corrected Student's t tests. **M**) Representative electron micrographs of body wall muscle blocks. Insets are representative cross-sections used for assessing myosin filament density. Scale bars (main images): 1 µm; Scale bars (insets): 100nm. **N**) Schematic of levamisole treatment contractibility assay. Images of worms were taken before and after levamisole treatment, and contractibility was defined as the % reduction in average worm length following treatment. **O**) Contractibility following levamisole treatment. n = 90 per condition; pooled from two biological replicates. Data are presented as mean ± SEM and analysed via two-way ANOVA with post-hoc FDR-corrected Students t tests. **P**) Touch-responsiveness. n = 90 per condition; pooled from three biological replicates. Data are presented as mean ± SEM and analysed via two-way ANOVA with post-hoc FDR-corrected Students t tests; ns, not significant ($P > 0.05$).
